# CT-Based Lesion Volume as an Independent Predictor of Surgical Recurrence in Medication-Related Osteonecrosis of the Jaw: A Multi-Center Study of 1007 Patients

**DOI:** 10.3390/jcm15145429

**Published:** 2026-07-10

**Authors:** Cheol Won Ryu, Sung Min Park, Hae Seo Park, Hui One Jung, Se Jin Han

**Affiliations:** Department of Oral and Maxillofacial Surgery, Dankook University Dental Hospital, Cheonan 31116, Republic of Korea; dbcjfdnjs10@dankook.ac.kr (C.W.R.); omspak@dankook.ac.kr (S.M.P.); 12120509@dankook.ac.kr (H.S.P.); jho.feb12@gmail.com (H.O.J.)

**Keywords:** medication-related osteonecrosis of the jaw, lesion volume, CT volumetry, surgical recurrence, risk stratification, multi-center study

## Abstract

**Background/Objectives**: To determine whether preoperative CT-measured lesion volume independently predicts surgical recurrence of MRONJ, and whether surgical approach influences recurrence after adjusting for lesion volume and clinical confounders. **Methods**: We conducted a retrospective multi-center cohort study of 1007 patients with single-lesion MRONJ who underwent surgery at five tertiary institutions in the Republic of Korea (2015–2023), with a minimum two-year follow-up. Preoperative lesion volume was measured by CT segmentation (Osteomyelitis Analyzer SW, version 2.0.6; Aemasue, Seoul, Republic of Korea) with cross-institutional consensus validation. Surgical recurrence was defined as re-fulfillment of 2022 AAOMS criteria after confirmed mucosal healing (Hayashida H1). Three logistic regression models of increasing adjustment were constructed a priori; reporting adhered to STROBE. **Results**: Recurrence occurred in 108 patients (10.7%) and increased in a dose–response pattern across volume quartiles (Q1 5.6%, Q2 9.2%, Q3 12.0%, Q4 16.0%; Cochran–Armitage trend *Z* = 3.89, *p* < 0.001), with a 2.9-fold relative gradient (absolute difference 10.4 percentage points). Surgeons preferentially selected aggressive approaches for larger lesions (OR = 1.651; 95% CI 1.338–2.037; *p* < 0.001). After adjustment, surgical approach was not an independent predictor in any model (fully adjusted OR = 0.735; 95% CI 0.381–1.419; *p* = 0.360), whereas lesion volume remained independent throughout (OR = 1.543–1.625 per log-unit; all *p* < 0.001; *c*-statistic 0.625–0.699). **Conclusions**: CT-measured preoperative lesion volume is an independent predictor of MRONJ surgical recurrence; we did not detect an independent surgical-approach effect, although the sample could not exclude a clinically meaningful one. Among measured factors, lesion volume—rather than surgical approach—was the principal predictor of recurrence risk, supporting consideration of routine CT volumetry in preoperative assessment, pending prospective validation.

## 1. Introduction

Medication-related osteonecrosis of the jaw (MRONJ) is a serious complication of antiresorptive therapy associated with significant morbidity and impaired quality of life [1,2]. Despite advances in surgical technique, recurrence following surgery remains a clinically important challenge, with reported rates of 10–30% across published series [3,4]. Identifying reliable preoperative predictors of recurrence would enable more precise risk stratification and individualized patient counseling.

In this context, the choice of surgical approach—conservative (sequestrectomy, saucerization) versus aggressive (decortication, resection)—has long been debated as a key determinant of recurrence. Some studies report superior outcomes with extensive surgical resection [5,6,7], while systematic reviews document heterogeneous outcomes that vary by stage and lesion characteristics [3,4], and consensus has not been reached. A common methodological limitation underlies these discrepant findings: it is expected that surgeons would preferentially select aggressive approaches for larger, more advanced lesions, which inherently carry higher recurrence risk. Without measuring and adjusting for lesion volume as the primary driver of this selection, any observed association between surgical approach and recurrence may reflect the severity of the underlying disease rather than a true treatment effect.

Computed tomography (CT)-based lesion volume measurement provides an objective, reproducible means of quantifying the actual lesion volume that directly influences surgical decision-making. Prior single-center studies have explored cone-beam CT (CBCT)-based volumetric segmentation of MRONJ lesions for surgical planning and prognostic assessment [8,9], but these cohorts comprised 78 and 67 patients, respectively, and did not formally adjust for confounding by indication between lesion volume and surgical aggressiveness. To our knowledge, no prior multi-center cohort has been sufficiently powered to disentangle the independent effects of preoperative lesion volume and surgical approach on recurrence. We, therefore, conducted a retrospective multi-center cohort study with two objectives: (1) to determine whether preoperative CT lesion volume independently predicts surgical recurrence and (2) to assess whether surgical approach independently influences recurrence after adjusting for lesion volume and clinical confounders. 

## 2. Materials and Methods

### 2.1. Study Design and Patients

This retrospective multi-center cohort study was conducted at five tertiary oral and maxillofacial surgery centers in the Republic of Korea (January 2015–December 2023), with reporting adhering to the Strengthening the Reporting of Observational Studies in Epidemiology (STROBE) guidelines. Institutional review board approval was obtained at all participating centers (approval number: DKUDH IRB 2023-04-006); informed consent was waived given the retrospective design and use of de-identified data.

Consecutive patients with MRONJ confirmed according to the 2022 American Association of Oral and Maxillofacial Surgeons (AAOMS) criteria who underwent surgery were screened [1]. Because a portion of the cohort was originally diagnosed prior to the 2022 AAOMS update (using the prevailing 2014 AAOMS criteria), all eligible records were retrospectively re-verified against the 2022 AAOMS diagnostic criteria during structured data abstraction by trained abstractors at each participating institution; patients whose original diagnosis did not satisfy the 2022 criteria were excluded at this stage. Inclusion criteria were as follows: (1) single-lesion MRONJ, operationally defined as the presence of a single contiguous osteonecrotic focus on preoperative CT, with the absence of a second anatomically discrete osteonecrotic lesion (defined as a separate focus located in a non-contiguous anatomical subunit—e.g., mandibular body versus ramus, or contralateral jaw quadrant—separated by radiographically normal bone) that would have required separate volumetric measurement; (2) available preoperative CT imaging suitable for segmentation-based volumetry; and (3) follow-up completion through December 2025, ensuring a minimum of two years for all patients. Patients managed with antibiotics alone, and patients with prior surgical management of the same MRONJ lesion, were excluded; patients with prior dental extractions or other dentoalveolar procedures unrelated to MRONJ surgery remained eligible.

### 2.2. CT-Based Lesion Volume Measurement

Manual segmentation of the osteonecrotic lesion was performed on consecutive axial CT slices at each participating institution using a single uniform segmentation software package across all five centers (Osteomyelitis Analyzer SW, version 2.0.6; Aemasue, Seoul, Republic of Korea). For each slice, the in-plane area of the segmented lesion was computed from the DICOM Pixel Spacing attribute (0028,0030), which specifies the physical width and height of a single pixel; the inter-slice distance was derived automatically by the software as the difference in the *z*-axis component of the Image Position (Patient) attribute (0020,0032) between adjacent slices, rather than relying on the nominal slice-thickness tag. Lesion volume (mm^3^) was then calculated as the sum, across all involved slices, of each segmented cross-sectional area multiplied by the corresponding inter-slice distance. A standardized segmentation protocol—including identical window settings, slice handling rules, and lesion-boundary definitions—was distributed to all participating centers prior to data collection to minimize between-center measurement variability. Because the cohort was assembled retrospectively across five centers, CT acquisition parameters (scanner model, slice thickness, and reconstruction kernel) were not prospectively standardized and varied across institutions. Two measures reduced the impact of this heterogeneity on volumetry. First, inter-slice distance was computed directly from the *z*-axis component of the DICOM Image Position (Patient) attribute (0020,0032) rather than from the nominal slice-thickness tag, so that the physical reconstruction interval—not the acquisition setting—governed the volume integration. Second, a shared diagnostic and labeling protocol specifying nine standardized radiographic criteria for identifying osteonecrotic bone on CBCT (bone destruction, sclerotic change, periosteal reaction, sequestrum formation, cortical disruption, soft-tissue involvement, radiolucent halo, pathological fracture, and consistency across contiguous slices; Supplementary Figure S1) was distributed to and applied at all participating centers, harmonizing lesion-boundary definitions independently of scanner differences. Residual variation in acquisition protocols is nonetheless acknowledged as a potential source of measurement error. To ensure cross-institutional labeling quality, a consensus-based validation protocol was implemented: each institution’s CT segmentations were independently reviewed by two or more examiners from a second participating institution, and cases judged to have inadequate segmentation boundaries were revised through collegial discussion until consensus was reached prior to volumetric calculation. Volume was log-transformed (natural logarithm) for statistical modeling. Quartile boundaries were defined as the 25th, 50th, and 75th percentiles of the lesion volume distribution in the full study cohort, yielding: Q1 < 1129 mm^3^ (*n* = 250), Q2 1129–2057 mm^3^ (*n* = 250), Q3 2058–4000 mm^3^ (*n* = 249), and Q4 > 4000 mm^3^ (*n* = 250); quartile analysis based on 999 patients with measured preoperative volume (8 of 1007 cohort patients had missing lesion-volume measurement and were excluded from quartile-specific analyses).

### 2.3. Outcome and Surgical Classification

The primary outcome was surgical recurrence. Healing was defined according to the Hayashida et al. classification as complete mucosal coverage of the operative site (H1 criterion) [10], assessed by the operating surgeon at standardized follow-up visits using identical criteria across all five participating institutions. Recurrence was defined as the re-fulfillment of 2022 AAOMS diagnostic criteria following confirmed mucosal healing: reappearance of exposed bone, or of bone that could be probed through an intraoral or extraoral fistula, or purulent discharge, at or adjacent to the operative site; or new radiographic evidence of osteonecrotic progression [1,11]. Consistent with the prevailing international definitions of MRONJ recurrence, recurrence events are typically identifiable within a one-year post-healing surveillance window [10]. The minimum two-year follow-up applied to all 1007 patients in the present cohort, therefore, confirms recurrence-free status well beyond this standard observation window, rather than introducing variability in observation time across patients. All patients completed structured clinical and radiographic follow-up through December 2025.

Surgical procedures were classified as follows: (1) conservative, i.e., sequestrectomy and/or saucerization, or (2) aggressive, i.e., decortication and/or segmental or marginal resection with or without reconstruction, based on the most invasive component performed. Decortication was grouped with resection as “aggressive” because both involve removal of bone extending beyond the macroscopically visible necrotic margin, in contrast to sequestrectomy and saucerization, which remove only macroscopically necrotic bone. This classification was applied uniformly across all participating institutions. When more than one procedure was recorded for a single patient (e.g., saucerization combined with decortication), classification was based on the most invasive procedure performed. Intravenous administration was operationally defined as any documented IV antiresorptive administration, including patients receiving combined IV plus oral regimens; this broad “any-IV-exposure” categorization was adopted because the recurrence-relevant biological mechanism (potent osteoclast inhibition and impaired bone remodeling) is initiated by any cumulative IV exposure regardless of concurrent oral administration. A small subset of patients with AAOMS Stage 0 disease (*n* = 13, 1.3% of the analytic cohort) were included because they underwent surgical management on a case-by-case basis for progressive non-specific symptoms or radiographic disease progression refractory to initial conservative pharmacologic management; a post hoc sensitivity analysis excluding these 13 patients yielded effect estimates virtually identical to the primary model (Δ in lesion-volume OR = 0.027, Δ in surgical-approach OR = 0.005, Δ in *c*-statistic = 0.006; Table A2).

### 2.4. Statistical Analysis

Three logistic regression models were constructed a priori to disentangle the independent effects of lesion volume and surgical approach: Model A (lesion volume alone, unadjusted); Model B (lesion volume plus surgical approach); Model C (fully adjusted). Candidate covariates for Model C were not selected empirically but were pre-specified on the basis of established pathophysiological pathways through which each factor may influence MRONJ healing and recurrence, organized along six conceptual domains: (i) lesion characteristics—preoperative CT lesion volume and anatomical location, the mandible being involved roughly twice as often as the maxilla owing to its denser cortical bone and more limited vascular supply; (ii) causative drug exposure—administration route (intravenous versus oral) and cumulative duration, higher-potency intravenous therapy over longer periods progressively suppressing bone turnover; (iii) surgical approach—conservative versus aggressive resection, the principal exposure under evaluation; (iv) patient factors—sex and age, recognized demographic risk modifiers; (v) systemic comorbidities with established pathophysiological links to MRONJ—diabetes mellitus (microvascular ischemia, impaired neutrophil function, and delayed wound healing), systemic corticosteroid use (suppressed bone formation and immunosuppression), malignancy (oncologic disease burden necessitating high-dose antiresorptive therapy, with concurrent chemotherapy subsumed under malignancy status given near-complete overlap), and immune disease—rheumatoid arthritis and other immune-related disorders—reflecting chronic immunosuppression; and (vi) local precipitating factors—dental surgical trauma (defined as the union of tooth extraction and other bone surgery), dental implant placement, and odontogenic infection, all recognized local triggers of jaw osteonecrosis. Mandibular location was defined as mandibular involvement, including both mandible-only and concurrent mandibular–maxillary disease. Systemic conditions lacking consistent pathophysiological evidence in MRONJ (e.g., hypertension, hyperlipidemia) were deliberately excluded from the adjustment set to avoid over-adjustment. Continuous variables (age in years, log-transformed lesion volume) were entered as continuous covariates; categorical and dichotomous variables (including drug duration dichotomized at ≥3 years versus <3 years, consistent with the categorical reporting threshold adopted across the MRONJ literature) were entered as binary indicators. AAOMS stage was not included as a covariate in the primary multivariable model because of its high overall missingness (23.8%) and the differential missingness pattern between recurrence and non-recurrence groups; given that AAOMS stage and lesion volume are conceptually overlapping descriptors of disease extent, the inclusion of CT volume as a continuous, objectively measured surrogate of disease extent was considered methodologically preferable to including a partially missing, categorically coarse, clinician-rated stage. In a sensitivity analysis restricted to the 767 patients with documented stage, adding AAOMS stage to Model C left the lesion-volume effect essentially unchanged (OR = 1.425; 95% CI 1.044–1.944; *p* = 0.025) and the surgical-approach estimate non-significant (OR = 0.922; *p* = 0.830), whereas stage itself was not an independent predictor (OR = 0.992; *p* = 0.969), supporting CT volume as the preferred continuous measure of disease extent. A complete description of all study variables, including operational definitions, coding, and missingness, is provided in Supplementary Table S1.

To characterize the relationship between lesion volume and surgical approach selection, a separate logistic regression was constructed with aggressive surgical approach as the binary outcome. Odds ratios (ORs) are reported with 95% confidence intervals (CIs) by the Wald method. Model calibration was assessed by the Hosmer–Lemeshow test. The dose–response relationship between volume quartile and recurrence rate was assessed by the Cochran–Armitage trend test [12]. To assess the pooled effect of surgical approach on recurrence across volume quartiles while accounting for the limited power of stratum-specific Fisher’s exact tests, a Cochran–Mantel–Haenszel (CMH) common odds ratio with Tarone’s test of homogeneity was additionally computed [13,14]. Categorical and continuous variables were compared by chi-square or Fisher’s exact test and Mann–Whitney U test, respectively.

Missing data (<6% for all covariates except systemic steroid use [10.0%], drug duration [12.3%], and AAOMS stage [23.8%]) were handled by single imputation in the primary multivariable analysis: binary covariates with missing values were coded as the absent (reference) category, and missing continuous covariates (log-transformed lesion volume and age) were imputed with the sample median, so that all 1007 patients contributed to Model C. Single imputation was pre-specified as the primary approach because missingness exceeded 10% for only three of the fourteen covariates (systemic steroid use, drug duration, and AAOMS stage), and an unambiguous, fully reproducible analytic dataset was prioritized for the primary analysis. We acknowledge that this strategy has a recognized limitation: coding missing binary covariates to the reference (absent) category assumes absence of the characteristic and can, therefore, bias the estimated effect of an incompletely recorded covariate toward the null. This concern is mitigated by two observations. First, all three covariates with >10% missingness were non-significant in both the primary and the multiple-imputation models, limiting their leverage on the principal conclusions. Second, the pre-specified multiple-imputation analysis (MICE, *m* = 20; Rubin’s rules; Table A4), which does not impose this reference-category assumption, yielded near-identical estimates for the two principal predictors (lesion-volume OR 1.543 vs. 1.539; surgical-approach OR 0.735 vs. 0.739; Δ *c*-statistic < 0.001), indicating that the single-imputation assumption did not materially distort effect estimation. Consistent with contemporary methodological guidance favoring multiple imputation, the concordance between the two approaches is the basis on which the single-imputation primary analysis is reported. A pre-specified sensitivity analysis using multiple imputation by chained equations (MICE) with *m* = 20 imputations and Rubin’s rules for combining estimates was additionally performed to validate the robustness of the Model C estimates against the single-imputation approach (Table A4). To further evaluate the robustness of the principal effect estimates, three additional sensitivity analyses were pre-specified: (i) non-parametric bootstrap percentile confidence intervals (1000 resamples) for the two principal predictors; (ii) Firth’s penalized-likelihood logistic regression [15], a bias-reduction method well suited to limited events-per-variable ratios; and (iii) a fixed-effect center-adjusted specification of Model C in which four center indicator variables were added to the covariate set (Table A5), evaluating the robustness of effect estimates against between-center variation in case mix and outcome rate.

All statistical analyses were performed using Python v3.12 (statsmodels v0.14, scipy v1.11); *p* < 0.05 was considered statistically significant. Given 108 recurrence events and 13 covariates in addition to lesion volume in Model C, the events-per-variable (EPV) ratio was 7.71, slightly below the conventional threshold of 10 for fully adjusted multivariable logistic regression; the stability of the principal lesion-volume effect estimate across Models A (no covariates), B (single covariate), and C (13 covariates)—yielding OR = 1.588, 1.625, and 1.543, respectively—serves as an internal sensitivity check confirming that the estimate is robust to the dimensionality of the covariate set. 

### 2.5. Sample Size and Power Considerations

This study used a retrospective consecutive-enrollment design; the available sample was, therefore, determined by the number of eligible MRONJ surgical cases across the five participating institutions during the study period (*n* = 1007 with 108 recurrence events), rather than by an a priori target. For the primary objective—the independent association between lesion volume and recurrence—108 events against 14 model terms (lesion volume plus 13 covariates) yielded an events-per-variable ratio of 7.71, and the stability of the lesion-volume estimate across Models A–C confirmed adequacy for this contrast. Because the surgical-approach comparison was a key secondary objective, a post hoc power analysis was performed under the observed design (131 aggressively managed patients, 12 events; 876 conservatively managed patients, 96 events; baseline conservative recurrence 11.0%). For the unadjusted two-proportion comparison, the study had approximately 58% power to detect a halving of recurrence (relative risk 0.50) and approximately 27% power to detect a one-third reduction (relative risk 0.67) at two-sided α = 0.05. For the fully adjusted odds ratio, simulation-based power (1000 replications) was approximately 48% to detect a true OR of 0.50, 28% for 0.60, and 11% for 0.75. Detecting a halving of recurrence odds at 80% power would have required approximately 221 aggressively managed patients. The surgical-approach comparison was, therefore, powered to detect only large effects and was interpreted as a secondary objective accordingly.

## 3. Results

### 3.1. Cohort Characteristics

**Figure 1:**
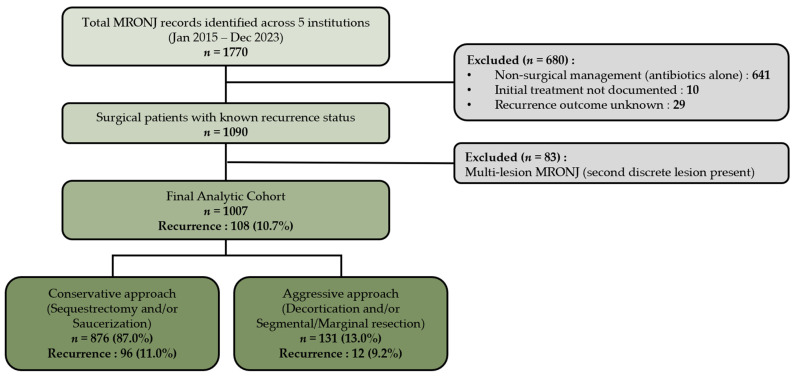
STROBE flow diagram showing patient selection cascade across five participating institutions. From the 1770 MRONJ records identified, 680 were excluded prior to multi-lesion assessment (641 managed with antibiotics alone; 10 with missing initial-treatment documentation; 29 with unknown recurrence outcome), leaving 1090 surgical patients with known recurrence status. A further 83 patients with multi-lesion MRONJ requiring separate volumetric measurement were excluded, yielding the final analytic cohort of 1007 patients (108 with surgical recurrence, 10.7%).

Of 1770 MRONJ records identified across the five participating institutions, 680 were excluded prior to multi-lesion assessment (641 managed with antibiotics alone without surgical intervention; 10 with missing initial-treatment documentation; 29 with unknown recurrence outcome), leaving 1090 surgical patients with known recurrence status. A total of 83 additional patients were excluded for multi-lesion MRONJ requiring separate volumetric measurement, yielding the final analytic cohort of 1007 patients (Figure 1). All patients completed a minimum two-year follow-up through December 2025. Surgical recurrence occurred in 108 patients (10.7%). Median age was 78 years (IQR 73–83); 70.3% had mandibular involvement. Intravenous administration was documented in 54.0%; drug duration was ≥3 years in 78.0% of patients with available records. Conservative surgical approach was performed in 876 (87.0%) and aggressive in 131 (13.0%). Patients with recurrence had significantly larger preoperative lesion volumes (median 3106 mm^3^ [IQR 1541–6000] vs. 1991 mm^3^ [IQR 1108–3869]; *p* < 0.001), higher rates of odontogenic infection (26.4% vs. 12.1%; *p* < 0.001), and dental surgical trauma as precipitating factor (63.1% vs. 52.9%; *p* = 0.049). All other characteristics, including surgical approach, did not differ significantly (Table 1).

### 3.2. Surgical Approach Selection by Lesion Volume

Median lesion volume was significantly larger in patients receiving an aggressive versus conservative surgical approach (3587 mm^3^ vs. 1912 mm^3^; *p* < 0.001). The rate of aggressive approach increased across volume quartiles: Q1 6.8%, Q2 8.4%, Q3 14.9%, and Q4 22.4%. Logistic regression identified lesion volume (OR = 1.651; 95% CI 1.338–2.037; *p* < 0.001), younger age (OR = 0.965; 95% CI 0.943–0.988; *p* = 0.003), and drug duration ≥ 3 years (OR = 1.868; 95% CI 1.186–2.943; *p* = 0.007) as independent determinants of surgical approach selection in the multivariable model (Table A3).

### 3.3. Lesion Volume and Recurrence: Dose–Response Analysis

**Figure 2:**
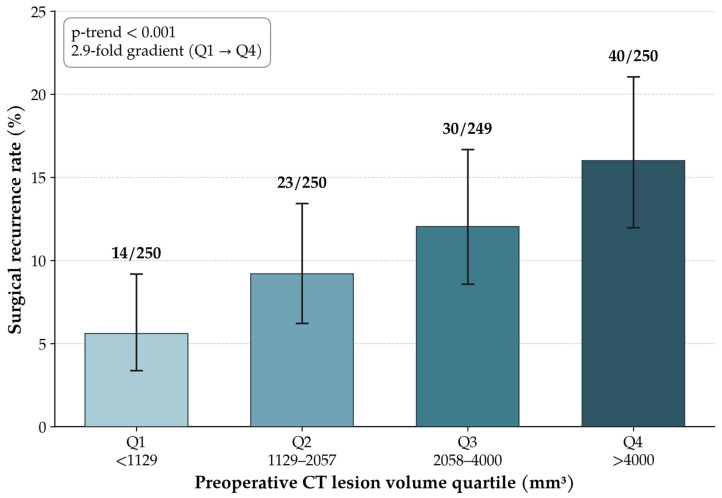
Recurrence rates by preoperative CT lesion volume quartile (*n* = 999). Quartile boundaries were defined as the 25th, 50th, and 75th percentiles of the lesion volume distribution: Q1 < 1129 mm^3^ (*n* = 250), Q2 1129–2057 mm^3^ (*n* = 250), and Q3 2058–4000 mm^3^ (*n* = 249), Q4 > 4000 mm^3^ (*n* = 250); 8 of the 1007 cohort patients had missing lesion-volume measurement and were excluded from this analysis. Recurrence increased progressively from Q1 5.6% to Q4 16.0% (Cochran–Armitage trend *Z* = 3.89; *p* < 0.001). Error bars: 95% Wilson confidence intervals. Numbers above bars: recurrence events/total patients per quartile.

Recurrence rates increased progressively across volume quartiles (Figure 2): Q1 5.6% (95% CI 3.4–9.2%), Q2 9.2% (6.2–13.4%), Q3 12.0% (8.6–16.7%), and Q4 16.0% (12.0–21.1%) (Cochran–Armitage trend *Z* = 3.89; *p* < 0.001). Lesion volume modeled as a continuous predictor was independently associated with recurrence across all three regression models (OR = 1.543–1.625 per log-unit; all *p* < 0.001; Table 2).

### 3.4. Surgical Approach and Recurrence: Within-Strata Analysis

**Figure 3:**
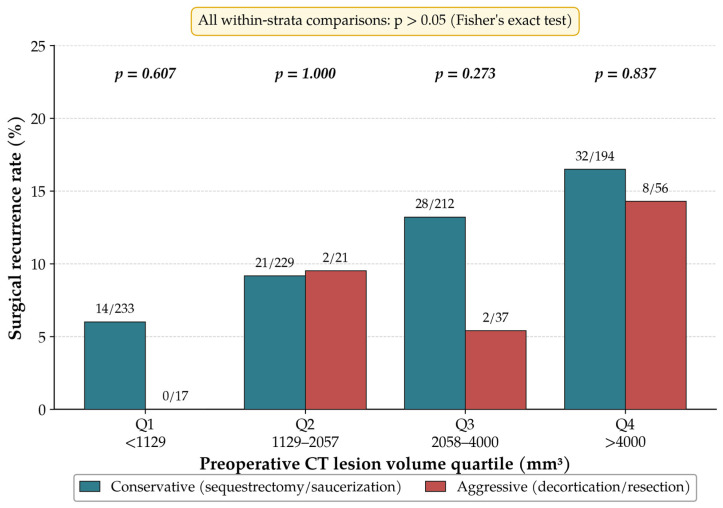
Recurrence rates by lesion volume quartile stratified by surgical approach. Within each quartile, no statistically significant difference was observed between conservative (sequestrectomy/saucerization; blue bars) and aggressive (decortication/resection; red bars) surgical approaches (all *p* > 0.05, Fisher’s exact test). Conservative: Q1–Q4, *n* = 233, 229, 212, and 194; aggressive: Q1–Q4, *n* = 17, 21, 37, and 56. *p*-values for Q1 and Q2 strata should be interpreted with caution due to small aggressive subgroup sizes (*n* = 17 and *n* = 21); no recurrence events were observed in the Q1 aggressive subgroup.

Recurrence rates did not differ significantly between conservative and aggressive surgical approaches within any volume quartile (Figure 3). Because aggressively managed patients were sparse in the lower quartiles (Q1 and Q2, *n* = 17 and *n* = 21), stratum-specific comparisons were underpowered and are not individually reported; the association was instead evaluated by a pooled analysis. The proportion receiving an aggressive approach was similar among patients who recurred (11.1%) and those who did not (13.2%; *p* = 0.535). A pooled Cochran–Mantel–Haenszel analysis across all four volume strata yielded a common odds ratio for the aggressive versus conservative approach of 0.659 (95% CI 0.345–1.260; *p* = 0.208), with no evidence of effect-measure heterogeneity across strata (Tarone’s homogeneity test, *p* = 0.578). The point estimates within Q3 (an eight percentage-point absolute reduction in the aggressive arm) and Q1 (zero recurrences in 17 aggressive cases) reflect uncertainty rather than evidence of difference, and a clinically meaningful effect cannot be excluded at the available sample size.

### 3.5. Multivariate Logistic Regression

Across all three pre-specified models, lesion volume was an independent predictor of recurrence (OR = 1.588–1.625 in Models A–B; OR = 1.543 in Model C; all *p* < 0.001), while surgical approach was not a statistically significant predictor in any model (Model A: OR = 0.819, *p* = 0.536; Model B: OR = 0.660, *p* = 0.204; Model C: OR = 0.735, *p* = 0.360; Table 2). Additional independent predictors in Model C were odontogenic infection (OR = 3.335; 95% CI 1.856–5.992; *p* < 0.001), dental surgical trauma (OR = 2.010; 95% CI 1.203–3.359; *p* = 0.008), and female sex, which was associated with lower recurrence odds (OR = 0.477; 95% CI 0.239–0.954; *p* = 0.036). The Hosmer–Lemeshow test indicated adequate calibration of Model C (*p* = 0.915); model discrimination assessed by the *c*-statistic (area under the receiver-operating-characteristic curve) was 0.699.

## 4. Discussion

This multi-center study of 1007 patients with single-lesion MRONJ, all followed for a minimum of two years, makes three inter-related contributions. First, it demonstrates a monotonic dose–response relationship between preoperative CT lesion volume and surgical recurrence, with rates ranging from 5.6% to 16.0% across quartiles—a 2.9-fold relative difference (absolute difference 10.4 percentage points)—consistently confirmed across all three regression models. To our knowledge, this represents the largest multi-center cohort to date applying CT-based volumetry to MRONJ surgical recurrence, with a sample size more than 12-fold greater than the largest prior single-center reports (*n* = 78 and *n* = 67) [8,9]. Second, it formally quantifies the selection pattern hypothesized in the introduction: surgeons do preferentially select aggressive approaches for larger lesions (OR = 1.651; *p* < 0.001), confirming the confounding by indication that motivated our multi-model design. Third, after accounting for this confounding, surgical approach did not emerge as a statistically significant independent predictor of recurrence in any of the three regression models or within any volume stratum. We emphasize that this represents a failure to detect an independent association at the available sample size rather than positive evidence that the two approaches yield equivalent recurrence risk.

Prior single-arm series reporting superior outcomes after resective surgery [5,6,7,16] drew predominantly from advanced-stage cohorts in which larger lesions were preferentially treated aggressively, without formal adjustment for indication bias. Systematic reviews [3,4] have documented heterogeneous outcomes that vary by disease stage and lesion characteristics, consistent with the confounding structure confirmed in this study. When larger lesions are simultaneously more likely to receive aggressive treatment and more likely to recur, crude comparisons conflate treatment effect with disease severity. Our multi-model design directly addresses this asymmetry: after adjustment for lesion volume, no independent association between surgical approach and recurrence was detected, identifying lesion biology as the principal measured predictor of outcome in this cohort; this should not be construed as establishing therapeutic equivalence between approaches.

The biological plausibility of lesion volume as a predictor of recurrence is well supported by MRONJ pathophysiology. A key mechanism is suppression of osteoclast-mediated bone remodeling: in the absence of normal remodeling, damaged bone cannot be replaced, perpetuating a biologically impaired environment beyond the margins of macroscopically visible necrosis [17]. Concurrently, antiresorptive agents suppress local angiogenesis by disrupting the physiological coupling between bone resorption and vascular remodeling, resulting in tissue ischemia that extends spatially with increasing disease burden [18]. Larger lesion volume, therefore, reflects a greater spatial extent of these pathophysiological changes—impaired vascularity, osteocyte necrosis, and biofilm colonization—that persist despite debridement of visible necrosis. This is directly supported by fluorescence-guided surgery studies, in which clinically viable-appearing bone at surgical margins is found to harbor histological evidence of osteonecrosis [19,20]. This biological framework helps explain why surgical approach selection alone may be insufficient to overcome recurrence risk determined by preoperative lesion extent.

The independent association of female sex with lower recurrence risk (OR = 0.477) was an unanticipated finding. In our cohort, recurrence occurred in 10.1% of women versus 19.7% of men, although the male subgroup was small (*n* = 66). This may reflect the predominantly osteoporosis-related (rather than oncologic) indication for antiresorptive therapy among women, who comprised 93.4% of the cohort, in contrast to a higher proportion of high-potency intravenous and oncologic exposure among men. We caution, however, that the mechanism remains uncertain and the estimate is based on a limited number of male patients; this observation should be regarded as hypothesis-generating and warrants confirmation in cohorts with more balanced sex distribution.

These findings have direct clinical implications. Patients in the highest volume quartile (>4000 mm^3^) face roughly threefold higher recurrence risk than those in the lowest, and this gradient did not appear to vary detectably by surgical approach in our cohort. Lesion volume is a statistically independent predictor of recurrence across all three models, with a strong, monotonic dose–response association; as a single marker, however, its discriminatory performance is moderate (*c*-statistic 0.625 for volume alone, rising to 0.699 in the fully adjusted model). These two properties are complementary rather than contradictory: a robust independent association does not by itself confer high individual-level discrimination. Accordingly, CT volumetry is best deployed as a key component of multivariable preoperative risk stratification rather than as a standalone prognostic test, identifying higher-risk patients who may benefit from intensified postoperative surveillance or adjuvant management and informing evidence-based patient counseling. Several practical applications follow from this framing. First, an objective preoperative estimate of recurrence risk could sharpen patient counseling, allowing the anticipated likelihood of a second episode to be communicated on the basis of measured disease burden rather than qualitative impression. Second, because recurrence risk increased steeply across volume quartiles, volumetric stratification could be used to calibrate the intensity of postoperative follow-up—for example, more frequent or longer clinical and radiographic surveillance for patients with high-volume lesions, who are the most likely to benefit from early detection of recurrence. Third, rather than displacing the established AAOMS staging system, CT volumetry is best regarded as a quantitative complement to it: staging captures the clinical and anatomical severity of disease, whereas continuous lesion volume adds an objective, reproducible measure of necrotic extent that is not constrained by the discrete boundaries of categorical stage. The moderate discrimination of the fully adjusted model (*c*-statistic 0.699) indicates that the measured covariates, while collectively informative, do not fully capture individual recurrence risk. Unmeasured determinants—antiresorptive drug class, drug-holiday duration, histopathological margin status, surgeon experience, and host-level biological factors—likely account for part of the residual unexplained variation, and their incorporation represents a clear avenue for improving future predictive models. More broadly, the moderate performance of volume as a single marker underscores that no individual variable is likely to predict MRONJ recurrence with high accuracy. Clinically useful prognostic models will probably need to integrate complementary classes of information—radiographic descriptors such as lesion volume and topography, clinical and demographic factors, pharmacological exposure including antiresorptive class and cumulative dose, surgical variables, and emerging biological or molecular markers—so that the limited discrimination contributed by any single domain is offset by the others. Lesion volume is best viewed as one robust, objective input to such a multidimensional model rather than as a sufficient predictor in isolation. Our findings do not argue against aggressive surgery per se; rather, they indicate that once preoperative lesion volume is accounted for, no independent effect of surgical approach on recurrence was detected. As resection to macroscopically healthy bone margins is the accepted surgical standard, the aggressive approaches analyzed were performed in accordance with this principle.

Several limitations should be acknowledged. The retrospective design precludes control of unmeasured confounders and relies on chart-abstracted exposure data: drug holiday duration, surgeon experience, and histopathological margin status could not be reliably ascertained, and the specific class and agent of antiresorptive therapy were not recorded and, therefore, could not be adjusted for. This is a non-trivial limitation: bisphosphonates bind avidly to bone mineral and exert a prolonged skeletal effect that persists after discontinuation, whereas denosumab acts through reversible RANKL inhibition with a substantially shorter biological half-life, and these divergent pharmacokinetic and pharmacodynamic profiles may plausibly influence healing capacity and recurrence risk. The administration route and cumulative duration adjusted for in our models capture only part of this exposure heterogeneity, and residual confounding by drug class cannot be excluded. Stratified analysis by antiresorptive agent should be a priority in future prospective cohorts. Systemic corticosteroid use was documented at a low positive frequency (14 of 906 patients with available data, 1.5%); although retained as an adjustment covariate in Model C on pathophysiological grounds, its low positive count yields a wide confidence interval (OR = 0.691; 95% CI 0.081–5.867), and residual confounding cannot be excluded. The principal effect estimates were nonetheless stable to this sparsity: Firth’s penalized-likelihood regression, which is robust to low events-per-variable ratios and rare-covariate separation, reproduced the lesion-volume and surgical-approach estimates almost identically (lesion-volume OR = 1.528; surgical-approach OR = 0.763), indicating that retention of this rare covariate did not materially destabilize the model.

Lesion volume was measured by manual segmentation. To minimize inter-observer variability, a standardized nine-criteria labeling protocol and a cross-institutional consensus-review process were applied prospectively to the segmentation workflow (Section 2.2). Because the retrospective design did not generate repeated independent measurements of identical lesions, formal reproducibility indices (intraclass correlation or Dice similarity coefficients) were not computed; as lesion volume is the primary exposure, any residual non-differential measurement error would be expected to bias the volume–recurrence association toward the null, so the observed association is, if anything, likely to be conservative. Outcome adjudication was non-blinded; any resulting bias would most plausibly act against a true surgical-approach effect. The manual segmentation used here, while reproducible under the standardized protocol applied across centers, is relatively time-consuming and requires dedicated software and operator expertise, which may limit its routine adoption in everyday clinical workflows. Recent progress in semi-automated and deep-learning-based segmentation of maxillofacial bone lesions suggests that much of this burden could be reduced in the near future: automated or operator-supervised algorithms could deliver lesion-volume estimates rapidly and with high reproducibility, lowering the expertise barrier and making volumetric risk stratification practical at the point of care. Prospective validation of such automated pipelines against expert manual segmentation will be an important step toward broader clinical implementation of CT volumetry in MRONJ.

Center-level random effects were not modeled given the small case volumes at two centers (*n* = 39 and *n* = 46); a fixed-effect center-adjusted model (Table A5) yielded principal estimates consistent with the primary analysis (lesion-volume OR Δ = +0.023, *p* < 0.001; surgical approach non-significant; *c*-statistic 0.708). The surgical-approach contrast was further limited by the small number of aggressively managed patients (*n* = 131, 12 events), within both the lower volume strata (Q1 and Q2, *n* = 17 and *n* = 21) and the cohort overall; the surgical-approach analysis was powered to detect only large effects (approximately 48% power for a true odds ratio of 0.50), so the null estimate reflects an absence of detectable association. The consistency of this null across the pooled Cochran–Mantel–Haenszel estimate, Tarone’s homogeneity test, and the non-parametric bootstrap nonetheless argues against a large, missed effect. A further consideration is that the binary “conservative” versus “aggressive” classification, although operationally defined by the most invasive component performed, collapses a broad spectrum of distinct procedures within each category—sequestrectomy and saucerization on the one hand, and decortication, marginal resection, and segmental resection with or without reconstruction on the other. These procedures differ in the extent of bone removed and in the biological aggressiveness of margin clearance, and the residual heterogeneity contained within each dichotomous category may have reduced our ability to detect outcome differences attributable to specific surgical techniques. A more granular procedure-level analysis would require a substantially larger number of events per technique than was available here and represents a worthwhile direction for future multi-center study. To retain the full sample, missing covariates were handled by single imputation; multiple imputation by chained equations (Table A4) gave essentially identical estimates (Δ in principal odds ratios ≤ 0.005), as did non-parametric bootstrap (1000 resamples; lesion-volume 95% CI 1.252–1.982; surgical-approach 95% CI 0.314–1.310), confirming robustness despite the limited events-per-variable ratio. 

The exact calendar date of recurrence was not consistently recorded across the participating institutions during structured data abstraction, and time-to-recurrence could, therefore, not be reconstructed for survival analysis. Because all patients completed a uniform minimum two-year observation by inclusion design—well beyond the conventional one-year post-healing surveillance window during which MRONJ recurrences are typically identified [10]—the binary recurrence endpoint captures recurrence-free status across a homogeneous and clinically sufficient follow-up interval, and the loss of information from not modeling event timing is correspondingly limited. We nonetheless acknowledge that time-to-event analysis could have provided additional insight into the tempo of recurrence (e.g., whether larger lesions recur earlier), and the incorporation of recurrence timing represents a valuable direction for prospective study. Under the present uniform-follow-up design with no observed deaths and no differential censoring, logistic regression rather than time-to-event modeling was the methodologically appropriate analytic framework.

Finally, patients managed with antibiotics alone (*n* = 641) were excluded by design, as the study was framed to evaluate predictors of surgical recurrence rather than the broader question of conservative pharmacologic versus surgical management; because patients selected for surgery may differ systematically from those treated medically, the findings should be interpreted within the surgical-cohort context. All patients were Korean and treated at tertiary referral centers, so external validation in independent, multi-ethnic populations is warranted, and the dichotomous surgical classification may not map directly onto procedural categorizations used in every healthcare system. Because antiresorptive prescribing patterns, the distribution of underlying indications, surgical practice, and the organization of follow-up care differ across countries and ethnic groups, the volume–recurrence relationship and the cohort-derived quartile boundaries observed in this single-nation cohort cannot be assumed to transfer directly to other populations. We, therefore, regard prospective international validation—in cohorts with different ethnic backgrounds and healthcare systems—as an essential next step before CT volumetry can be adopted as a generalizable preoperative risk-stratification tool. The volume quartile boundaries reported here (Q1 < 1129/Q2 1129–2057/Q3 2058–4000/Q4 > 4000 mm^3^) were derived from the distribution of the present cohort and are intended solely to illustrate the dose–response gradient; they do not constitute validated clinical cut-off values and should not be applied as decision thresholds. Establishment of clinically actionable volumetric thresholds will require prospective validation in independent, demographically distinct cohorts.

## 5. Conclusions

In this multi-center cohort of single-lesion MRONJ, preoperative CT-measured lesion volume was an independent, dose–dependent predictor of surgical recurrence, whereas surgical approach showed no detectable independent effect once lesion volume was accounted for. Clinically, these findings reframe preoperative risk assessment around objective disease burden rather than the choice of surgical aggressiveness alone: routine CT volumetry could help identify higher-risk patients who may benefit from intensified postoperative surveillance, adjunctive measures, and individualized counseling, while the absence of a demonstrated surgical-approach effect—within a sample underpowered to exclude a clinically relevant one—argues for caution before assuming that more extensive resection necessarily lowers recurrence. Lesion volume should be integrated as one component of multivariable risk stratification rather than used as a standalone test, and the present cohort-derived thresholds require prospective validation in independent, demographically distinct populations before clinical application.

## Figures and Tables

**Table 1:** Baseline characteristics of patients with and without surgical recurrence (*n* = 1007).

Variable	Total (*n* = 1007)	No Recurrence (*n* = 899)	Recurrence (*n* = 108)	*p*-Value
Age, years, median (IQR)	78 (73–83)	78 (73–82)	80 (74–83)	0.165
MRONJ stage (AAOMS 2022) †	767 (76.2%)	694 (77.2%)	73 (67.6%)	0.328
Stage 0	13 (1.3%)	10 (1.1%)	3 (2.8%)	
Stage 1	73 (7.2%)	68 (7.6%)	5 (4.6%)	
Stage 2	494 (49.1%)	446 (49.6%)	48 (44.4%)	
Stage 3	187 (18.6%)	170 (18.9%)	17 (15.7%)	
Not documented	240 (23.8%)	205 (22.8%)	35 (32.4%)	
Preoperative lesion volume (mm^3^)	2057 (1129–4000)	1991 (1108–3869)	3106 (1541–6000)	<0.001
Q1 (<1129 mm^3^)	250 (25.0%)	236 (26.5%)	14 (13.1%)	<0.001
Q2 (1129–2057 mm^3^)	250 (25.0%)	227 (25.4%)	23 (21.5%)	
Q3 (2058–4000 mm^3^)	249 (24.9%)	219 (24.6%)	30 (28.0%)	
Q4 (>4000 mm^3^)	250 (25.0%)	210 (23.5%)	40 (37.4%)	
Mandibular location	708 (70.3%)	628 (69.9%)	80 (74.1%)	0.365
Intravenous administration	531 (54.0%)	484 (54.9%)	47 (45.6%)	0.073
Drug duration ≥ 3 years	689 (78.0%)	618 (78.1%)	71 (77.2%)	0.834
Aggressive surgical approach	131 (13.0%)	119 (13.2%)	12 (11.1%)	0.535
Sex (Female)	941 (93.4%)	846 (94.1%)	95 (88.0%)	0.015
Diabetes mellitus	271 (28.2%)	242 (28.1%)	29 (29.6%)	0.752
Systemic steroid use	14 (1.5%)	13 (1.6%)	1 (1.0%)	1.000
Malignancy	73 (7.6%)	63 (7.3%)	10 (10.2%)	0.305
Immune disease	94 (9.8%)	87 (10.1%)	7 (7.1%)	0.353
Dental surgical trauma (Extraction or Bone Surgery)	538 (54.0%)	473 (52.9%)	65 (63.1%)	0.049
Dental implant placement	231 (23.3%)	214 (24.0%)	17 (16.7%)	0.095
Odontogenic infection	136 (13.7%)	108 (12.1%)	28 (26.4%)	<0.001

Data are median (IQR) for continuous variables and n (%) for categorical variables. Statistical tests: Mann–Whitney U test for continuous variables; chi-square or Fisher’s exact test for categorical variables. Bold values indicate *p* < 0.05. IQR, interquartile range. † Stage was documented in 767 of 1007 patients (76.2%); the chi-square *p*-value compares recurrence across documented stages (Stages 0–3) only. Quartile boundaries were defined by the 25th, 50th, and 75th percentiles of the lesion volume distribution among the 999 patients with measured preoperative volume; 8 patients had missing volume measurements and were excluded from quartile-specific analyses. Subgroup counts in the No recurrence and Recurrence columns may not sum to the Total column where covariates contained missing values; percentages are calculated among patients with available data (i.e., the denominator for each covariate is the number of non-missing observations, not the full cohort of 1007). Numbers of patients with missing covariate data, and the corresponding available-data denominators (1007 minus missing), were as follows: intravenous administration 23 missing (*n* = 984), drug duration 124 missing (*n* = 883), dental surgical trauma 10 missing (*n* = 997), dental implant 15 missing (*n* = 992), odontogenic infection 12 missing (*n* = 995), diabetes mellitus 47 missing (*n* = 960), malignancy 47 missing (*n* = 960), immune disease 46 missing (*n* = 961), and systemic steroid use 101 missing (*n* = 906). Surgical approach classification: aggressive = decortication and/or segmental or marginal resection; conservative = sequestrectomy and/or saucerization.

**Table 2:** Logistic regression analysis of predictors of surgical recurrence: three a priori models.

Variable	Model A		Model B		Model C	
	OR	95% CI (*p*)	OR	95% CI (*p*)	OR	95% CI (*p*)
Lesion volume †	1.588	1.279–1.972 (<0.001)	1.625	1.305–2.023 (<0.001)	1.543	1.226–1.942 (<0.001)
Aggressive surgical approach	0.819	0.436–1.539 (0.536)	0.660	0.347–1.254 (0.204)	0.735	0.381–1.419 (0.360)
Odontogenic infection	—	—	—	—	3.335	1.856–5.992 (<0.001)
Dental surgical trauma (Extraction or Bone Surgery)	—	—	—	—	2.010	1.203–3.359 (0.008)
Sex (female)	—	—	—	—	0.477	0.239–0.954 (0.036)
Age, Mandibular location, IV route, Drug duration, Diabetes mellitus, Malignancy, Systemic steroid use, Immune disease, Dental implant placement	—	—	—	—	All NS	(see Table A1)

OR, odds ratio; CI, 95% confidence interval (Wald method). † Per log-unit increase in lesion volume (natural logarithm). Model A: unadjusted. Model B: lesion volume + surgical approach. Model C: fully adjusted. Hosmer–Lemeshow *p* = 0.915; *c*-statistic = 0.699 (Model C). NS, not significant. Detailed odds ratios, 95% confidence intervals, and *p*-values for the non-significant Model C covariates (age, mandibular location, intravenous administration, drug duration, diabetes mellitus, malignancy, systemic steroid use, and immune disease) are reported in Table A1.

## Data Availability

The data presented in this study are available from the corresponding author on reasonable request, subject to IRB restrictions.

## Supplementary Materials

The following supporting information can be downloaded at: https://www.mdpi.com/article/10.3390/jcm15145429/s1, Figure S1: Standardized CT segmentation and volumetric measurement workflow applied across all five participating institutions. Stage 1: a shared labeling protocol of nine predefined radiographic criteria for osteonecrotic bone was distributed to all participating institutions prior to data collection. Stage 2: the lesion was manually segmented on consecutive axial slices under identical window settings and lesion-boundary definitions, and the in-plane area of each slice was derived from the DICOM Pixel Spacing attribute (0028,0030). Stage 3: inter-slice distance was computed from the z-axis component of the Image Position (Patient) attribute (0020,0032) rather than the nominal slice-thickness tag, and lesion volume (mm^3^) was obtained as the sum across slices of each segmented cross-sectional area multiplied by the corresponding inter-slice distance, then natural-log-transformed. Stage 4: each center’s segmentations were independently reviewed by two or more examiners from a second institution and inadequate segmentation boundaries were revised through collegial discussion until consensus was reached prior to volumetric calculation; Table S1: Description of study variables: operational definitions, coding, and missingness in the analytic cohort (n = 1007).

## Appendix A

**Table A1:** Detailed odds ratios for non-significant covariates in Model C.

Covariate	OR	95% CI	*p*-Value
Age (per year)	1.012	0.985–1.040	0.381
Mandibular location	1.051	0.655–1.687	0.836
Intravenous administration	0.715	0.469–1.090	0.119
Drug duration ≥ 3 years	0.839	0.540–1.306	0.437
Diabetes mellitus	0.897	0.560–1.435	0.650
Malignancy	1.254	0.588–2.674	0.558
Immune disease	0.648	0.284–1.479	0.303
Systemic steroid use	0.691	0.081–5.867	0.735
Dental implant placement	1.092	0.591–2.017	0.779

OR, odds ratio; CI, confidence interval (Wald method). Estimates derived from the fully adjusted multivariable logistic regression model (Model C; *n* = 1007 with median imputation for missing continuous covariates and reference-category coding for missing binary covariates, 108 events) including lesion volume (log-transformed), surgical approach, odontogenic infection, dental surgical trauma (Extraction or Bone Surgery), mandibular location, sex, age, intravenous administration, drug duration (≥3 years vs. <3 years), diabetes mellitus, malignancy, systemic steroid use, immune disease, and dental implant placement. The covariates listed below were not significant predictors of recurrence (all *p* > 0.05).

**Table A2:** Sensitivity analysis: exclusion of AAOMS Stage 0 patients (*n* = 13).

Cohort	Lesion Volume OR (95% CI; *p*)	Surgical Approach OR (95% CI; *p*)	n	*c*-Statistic
Primary Model C (full cohort)	1.543 (1.226–1.942; <0.001)	0.735 (0.381–1.419; 0.360)	1007	0.699
Stage 0 excluded (*n* = 13)	1.570 (1.245–1.979; <0.001)	0.730 (0.378–1.409; 0.348)	994	0.693
Δ primary vs. sensitivity	0.027	0.005	—	0.006

Effect estimates for the two principal predictors (lesion volume and surgical approach) from the fully adjusted Model C, comparing the primary analysis (full analytic cohort, *n* = 1007) with a sensitivity analysis excluding 13 patients with AAOMS Stage 0 disease. The exclusion did not materially alter any estimate (Δ lesion-volume OR = 0.027, Δ surgical-approach OR = 0.005, Δ *c*-statistic = 0.006), confirming that the inclusion of Stage 0 surgical cases did not affect the principal findings of the study.

**Table A3:** Full multivariable logistic regression analysis of determinants of aggressive surgical approach selection (*n* = 1007).

Covariate	OR	95% CI	*p*-Value
Lesion volume (per log-unit)	1.651	1.338–2.037	**<0.001**
Age (per year)	0.965	0.943–0.988	**0.003**
Sex (Female)	1.229	0.547–2.761	0.617
Odontogenic infection	0.475	0.231–0.979	**0.044**
Dental surgical trauma (Extraction or Bone Surgery)	1.029	0.654–1.621	0.900
Mandibular location	1.165	0.759–1.789	0.484
Intravenous administration	0.784	0.534–1.153	0.217
Drug duration ≥ 3 years	1.868	1.186–2.943	**0.007**
Diabetes mellitus	0.876	0.566–1.355	0.552
Malignancy	1.233	0.597–2.549	0.572
Immune disease	1.389	0.779–2.477	0.266
Systemic steroid use	0.472	0.056–3.969	0.489
Dental implant placement	0.677	0.390–1.174	0.165

OR, odds ratio; CI, confidence interval (Wald method). Estimates from a multivariable logistic regression model with aggressive surgical approach (decortication and/or segmental or marginal resection) as the binary outcome (*n* = 1007, 131 events; median imputation for missing continuous covariates and reference-category coding for missing binary covariates). Lesion volume, younger age, and drug duration ≥ 3 years were the statistically significant determinants of surgical approach selection. Bold rows indicate *p* < 0.05.

**Table A4:** Sensitivity analysis: multiple imputation by chained equations (MICE, *m* = 20) versus single imputation (Model C, *n* = 1007).

Covariate	Single Imputation (Primary) OR (95% CI; *p*)	MICE (*m* = 20, Rubin’s Rules) OR (95% CI; *p*)
Lesion volume (per log-unit)	1.543 (1.226–1.942; <0.001)	1.539 (1.222–1.937; <0.001)
Aggressive surgical approach	0.735 (0.381–1.419; 0.360)	0.739 (0.383–1.424; 0.366)
Odontogenic infection	3.335 (1.856–5.992; <0.001)	3.425 (1.899–6.177; <0.001)
Dental surgical trauma (Extraction or Bone Surgery)	2.010 (1.203–3.359; 0.008)	2.160 (1.274–3.662; 0.004)
Mandibular location	1.051 (0.655–1.687; 0.836)	1.060 (0.660–1.702; 0.811)
Age (per year)	1.012 (0.985–1.040; 0.381)	1.012 (0.985–1.041; 0.379)
Sex (Female)	0.477 (0.239–0.954; 0.036)	0.472 (0.235–0.948; 0.035)
Intravenous administration	0.715 (0.469–1.090; 0.119)	0.753 (0.492–1.153; 0.192)
Drug duration ≥ 3 years	0.839 (0.540–1.306; 0.437)	0.907 (0.538–1.530; 0.714)
Diabetes mellitus	0.897 (0.560–1.435; 0.650)	0.969 (0.609–1.542; 0.894)
Malignancy	1.254 (0.588–2.674; 0.558)	1.310 (0.613–2.799; 0.485)
Immune disease	0.648 (0.284–1.479; 0.303)	0.693 (0.304–1.582; 0.384)
Systemic steroid use	0.691 (0.081–5.867; 0.735)	0.687 (0.079–5.972; 0.733)
Dental implant placement	1.092 (0.591–2.017; 0.779)	1.189 (0.639–2.213; 0.585)
Model *c*-statistic	0.699	0.698 (SD < 0.001 across imputations)

OR, odds ratio; CI, confidence interval. MICE was implemented using iterative chained equations with Bayesian ridge regression as the per-variable predictor, *m* = 20 imputations, posterior sampling enabled, and median initialization; binary covariates were thresholded at 0.5 post-imputation. Estimates were pooled using Rubin’s rules with Wald-based confidence intervals. All point estimates and statistical significance categories were preserved under MICE (Δ ≤ 0.005 in the two principal odds ratios for lesion volume and surgical approach; Δ *c*-statistic < 0.001), confirming the robustness of the Model C results to the choice of imputation strategy. The associations for odontogenic infection and dental surgical trauma remained statistically significant under both approaches (*p* < 0.001 and *p* < 0.01, respectively) and did not alter any clinical interpretation.

**Table A5:** Sensitivity analysis: fixed-effect center-adjusted Model C versus primary single-imputation Model C (*n* = 1007).

Covariate	Single Imputation (Primary) OR (95% CI; *p*)	Fixed-Effect Center-Adjusted OR (95% CI; *p*)
Lesion volume (per log-unit)	1.543 (1.226–1.942; <0.001)	1.566 (1.207–2.030; <0.001)
Aggressive surgical approach	0.735 (0.381–1.419; 0.360)	1.315 (0.608–2.846; 0.486)
Odontogenic infection	3.335 (1.856–5.992; <0.001)	3.277 (1.794–5.985; <0.001)
Dental surgical trauma (Extraction or Bone Surgery)	2.010 (1.203–3.359; 0.008)	1.913 (1.140–3.209; 0.014)
Mandibular location	1.051 (0.655–1.687; 0.836)	1.041 (0.647–1.675; 0.869)
Age (per year)	1.012 (0.985–1.040; 0.381)	1.013 (0.985–1.041; 0.361)
Sex (Female)	0.477 (0.239–0.954; 0.036)	0.486 (0.239–0.988; 0.046)
Intravenous administration	0.715 (0.469–1.090; 0.119)	0.691 (0.449–1.062; 0.092)
Drug duration ≥ 3 years	0.839 (0.540–1.306; 0.437)	0.910 (0.578–1.431; 0.682)
Diabetes mellitus	0.897 (0.560–1.435; 0.650)	0.924 (0.571–1.496; 0.749)
Malignancy	1.254 (0.588–2.674; 0.558)	1.306 (0.610–2.795; 0.492)
Immune disease	0.648 (0.284–1.479; 0.303)	0.844 (0.363–1.962; 0.693)
Systemic steroid use	0.691 (0.081–5.867; 0.735)	0.666 (0.074–6.006; 0.717)
Dental implant placement	1.092 (0.591–2.017; 0.779)	1.069 (0.576–1.984; 0.833)
*Center indicators* *(vs. Boramae Medical Center)*		
Boramae Medical Center (*n* = 39, recurrence 10.3%)	—	reference
Chosun University Dental Hospital (*n* = 410, recurrence 10.2%)	—	1.221 (0.387–3.851; 0.734)
Dankook University Dental Hospital (*n* = 239, recurrence 14.6%)	—	1.165 (0.357–3.801; 0.800)
Korea University Hospital (*n* = 46, recurrence 21.7%)	—	2.068 (0.536–7.985; 0.292)
Wonkwang University Dental Hospital (*n* = 273, recurrence 6.2%)	—	0.481 (0.138–1.682; 0.252)
Model *c*-statistic	0.699	0.708

OR, odds ratio; CI, confidence interval (Wald method). The fixed-effect center-adjusted model includes all 14 Model C covariates plus four center indicator variables, with Boramae Medical Center (the smallest analytic-cohort center) as the reference. All statistically significant Model C predictors (lesion volume, odontogenic infection, and dental surgical trauma) preserved their significance and effect direction under center adjustment, with modest point-estimate shifts. The point estimate for surgical approach varied in magnitude (Δ OR = +0.580) but remained statistically non-significant (*p* = 0.486), consistent with the primary finding that surgical approach was not independently associated with recurrence. Model *c*-statistic was marginally higher in the center-adjusted specification (0.708 vs. 0.699); although no individual center indicator reached statistical significance (all *p* > 0.25), their joint inclusion left the principal effect estimates for lesion volume and surgical approach consistent with the primary specification.
