# Supplementary material for: CT-Based Lesion Volume as an Independent Predictor of Surgical Recurrence in Medication-Related Osteonecrosis of the Jaw: A Multi-Center Study of 1007 Patients
Source: J Clin Med. 2026 Jul 10;15(14):5429. doi: 10.3390/jcm15145429 (PMC13412846; doi:10.3390/jcm15145429)
Supplement: Supplementary file 1 [file jcm-15-05429-s001.zip › jcm-4390101-supplementary.pdf]

Supplementary Materials

Table S1. Description of study variables: operational definitions, coding, and missingness in the analytic cohort (n = 1007).

Variable	Operational definition	Coding	Missing, n (%)
Lesion volume (per log-unit)	Preoperative CT-segmented lesion volume (mm^3), natural-log-transformed	Continuous	8 (0.8)
Mandibular location	Mandibular involvement, including both mandible-only and concurrent mandibular–maxillary disease	0 = no, 1 = yes	0 (0.0)
Intravenous administration	Any documented intravenous antiresorptive administration, including patients receiving combined intravenous plus oral regimens	0 = no, 1 = yes	23 (2.3)
Drug duration ≥3 years	Cumulative antiresorptive treatment duration of 3 years or longer	0 = no, 1 = yes	124 (12.3)
Aggressive surgical approach	Decortication or segmental/marginal resection, versus conservative treatment (sequestrectomy or saucerization)	0 = no, 1 = yes	0 (0.0)
Sex (Female)	Patient sex	0 = male, 1 = female	0 (0.0)
Age (per year)	Age at surgery (years)	Continuous	0 (0.0)
Dental surgical trauma (Extraction or Bone Surgery)	Union of tooth extraction and other bone surgery as a local precipitating factor	0 = no, 1 = yes	10 (1.0)
Dental implant placement	Dental implant placement as a local precipitating factor	0 = no, 1 = yes	15 (1.5)
Odontogenic infection	Odontogenic infection as a local precipitating factor	0 = no, 1 = yes	12 (1.2)
Diabetes mellitus	Diabetes mellitus	0 = no, 1 = yes	47 (4.7)
Systemic steroid use	Systemic corticosteroid use	0 = no, 1 = yes	101 (10.0)
Malignancy	Malignancy	0 = no, 1 = yes	47 (4.7)
Immune disease	Rheumatoid arthritis and other immune-related disorders	0 = no, 1 = yes	46 (4.6)
AAOMS stage [a]	2022 AAOMS disease stage at presentation	0, 1, 2, 3	240 (23.8)

The 14 variables listed above the rule constitute the covariate set of the fully adjusted model (Model C). Missingness is reported for the final analytic cohort of 1007 patients. In the primary analysis, missing continuous covariates (log-transformed lesion volume and age) were imputed with the sample median and missing binary covariates were coded as the absent (reference) category; a pre-specified multiple-imputation analysis (MICE, m = 20, with Rubin's rules) was performed as a sensitivity analysis. [a] AAOMS stage was not included among the Model C covariates because of its high missingness and its conceptual overlap with lesion volume as a descriptor of disease extent; it was used only in a sensitivity analysis restricted to the 767 patients with a documented stage. AAOMS, American Association of Oral and Maxillofacial Surgeons; CT, computed tomography; MICE, multiple imputation by chained equations.

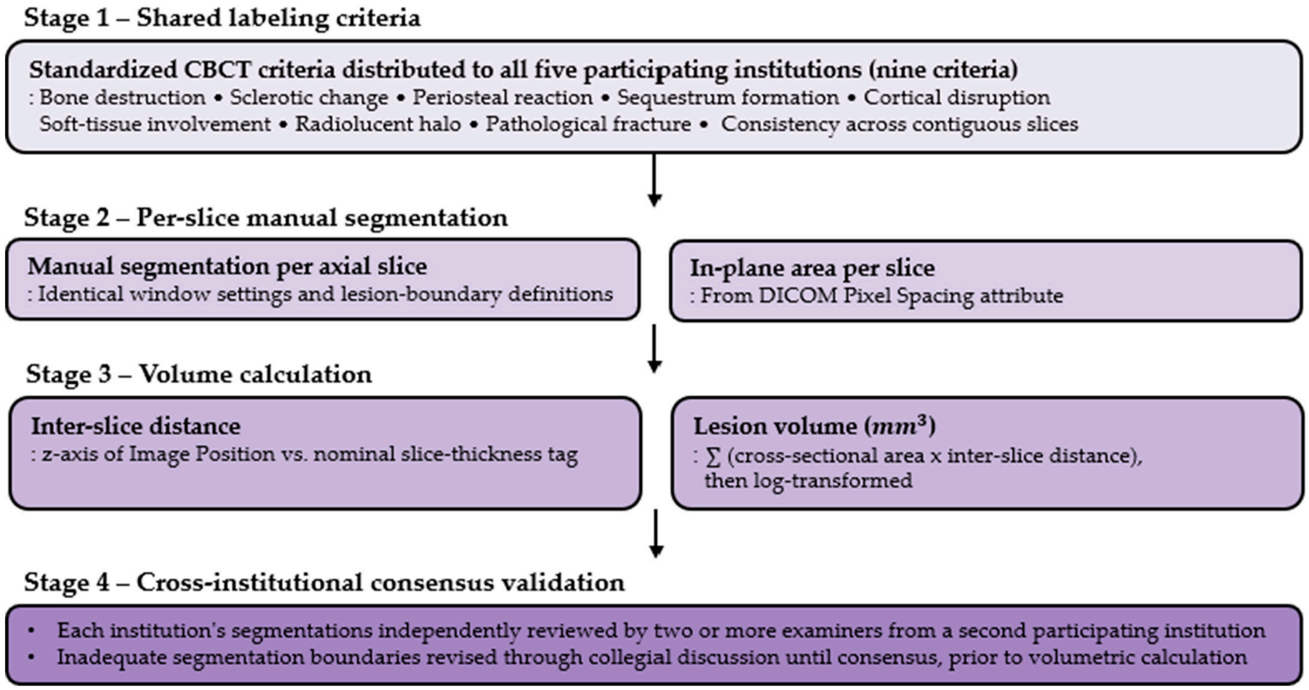

Figure S1. Standardized CT segmentation and volumetric measurement workflow applied across all five participating institutions. Stage 1: a shared labeling protocol of nine predefined radiographic criteria for osteonecrotic bone was distributed to every center. Stage 2: the lesion was manually traced on each axial slice under identical window settings, and the in-plane area was derived from the DICOM Pixel Spacing attribute (0028,0030). Stage 3: inter-slice distance was computed from the z-axis component of the Image Position (Patient) attribute (0020,0032), and lesion volume (mm3) was obtained as the sum across slices of area × inter-slice distance, then natural-log-transformed. Stage 4: each center's segmentations were independently reviewed by two or more examiners from a second institution and revised by consensus before volumetric calculation.
